# Salicylic Acid Is Involved in Rootstock–Scion Communication in Improving the Chilling Tolerance of Grafted Cucumber

**DOI:** 10.3389/fpls.2021.693344

**Published:** 2021-06-24

**Authors:** Xin Fu, Yi-Qing Feng, Xiao-Wei Zhang, Yan-Yan Zhang, Huan-Gai Bi, Xi-Zhen Ai

**Affiliations:** State Key Laboratory of Crop Biology, Key Laboratory of Crop Biology and Genetic Improvement of Horticultural Crops in Huanghuai Region, College of Horticulture Science and Engineering, Shandong Agricultural University, Tai’an, China

**Keywords:** cold-responsive genes, *Cucumis sativus*, *Cucurbita moschata*, grafting, salicylic acid, root–shoot communication

## Abstract

Salicylic acid (SA) has been proven to be a multifunctional signaling molecule that participates in the response of plants to abiotic stresses. In this study, we used cold-sensitive cucumber and cold-tolerant pumpkin as experimental materials to examine the roles of SA in root–shoot communication responses to aerial or/and root-zone chilling stress in own-root and hetero-root grafted cucumber and pumpkin plants. The results showed that pumpkin (*Cm*) rootstock enhanced the chilling tolerance of grafted cucumber, as evidenced by the observed lower levels of electrolyte leakage (EL), malondialdehyde (MDA), and higher photosynthetic rate (Pn) and gene expression of Rubisco activase (RCA). However, cucumber (*Cs*) rootstock decreased the chilling tolerance of grafted pumpkins. *Cs/Cm* plants showed an increase in the mRNA expression of C-repeat-binding factor (*CBF1*), an inducer of *CBF* expression (*ICE1*), and cold-responsive (*COR47*) genes and *CBF1* protein levels in leaves under 5/25 and 5/5°C stresses, or in roots under 25/5 and 5/5°C stresses, respectively, compared with the *Cs/Cs*. Chilling stress increased the endogenous SA content and the activity of phenylalanine ammonia-lyase (PAL), and the increase in SA content and activity of PAL in *Cs/Cm* plants was much higher than in *Cs/Cs* plants. Transcription profiling analysis revealed the key genes of SA biosynthesis, *PAL*, *ICS*, and *SABP2* were upregulated, while *SAMT*, the key gene of SA degradation, was downregulated in *Cs/Cm* leaves, compared with *Cs/Cs* leaves under chilling stress. The accumulation of SA in the *Cs/Cm* leaves was mainly attributed to an increase in SA biosynthesis in leaves and that in transport from roots under aerial and root-zone chilling stress, respectively. In addition, exogenous SA significantly upregulated the expression level of cold-responsive (*COR*) genes, enhanced actual photochemical efficiency (*Φ*_PSII_), maximum photochemical efficiency (*F*_v_/*F*_m_), and Pn, while decreased EL, MDA, and CI in grafted cucumber. These results suggest that SA is involved in rootstock–scion communication and grafting-induced chilling tolerance by upregulating the expression of *COR* genes in cucumber plants under chilling stress.

## Introduction

Cucumbers (*Cucumis sativus* L.) are cold-sensitive plants, and they often suffer from low temperatures during winter in solar greenhouses in northern China (Wu et al., 2016). Cold stress is considered an important constraint on crop growth and productivity in global agriculture because of its adverse effects on various physiological and metabolic processes of plant growth (Theocharis et al., 2012). Therefore, the mechanisms about the chilling stress responses in plants and the increase in the chilling tolerance of protected vegetables have recently attracted considerable attention.

Salicylic acid (SA), a plant hormone, has been widely regarded as a signal molecule that plays an important role in showing the response to biotic stress, which mediates the defense mechanism against pathogens and develops systemic acquired resistance (SAR; Dempsey et al., 2011; Metraux et al., 1990). Evidence is also increasing that application of SA enhances tolerance against abiotic stresses, such as heat, cold, drought, and salt stresses (Khan et al., 2015). In general, abiotic stresses induce an improved level of SA biosynthesis, helping to activate various other defensive compounds (Davies, 2004). SA can promote the tolerance of a plant to biotic and abiotic stress conditions by activating complex signal transduction cascades (Sendon et al., 2011; Shin et al., 2018). For example, the application of exogenous SA improved chilling/freezing tolerance as evidenced by reduced accumulation of excess hydrogen peroxide (H_2_O_2_), reduced leakage of ions, and alleviated oxidative stress (Dong et al., 2014; Shin et al., 2018). Nitric oxide (NO) produced by SA upregulated the activities of the ascorbate–glutathione (AsA-GSH) cycle and antioxidant enzymes so that it could play a central function as a signaling molecule in the salt tolerance of pepper plants (Kaya et al., 2020).

Cold acclimation is one of the major mechanisms by which plants adapt to cold stress by activating a set of cold-responsive (*COR*) genes, which encode cryoprotective proteins to alleviate the damage of plant cells caused by chilling stress (Thomashow, 1999). The ICE1-CBF-COR transcriptional cascade is generally considered as the best understood cold acclimation signaling pathway. In this pathway, C-repeat binding factors (CBFs) are crucial transcription factors (Zhou et al., 2018), and they are essential for the induction of chilling tolerance (Hannah et al., 2006; Kang et al., 2013). Thomashow (2001) reported that overexpression of *CBF* genes in *Arabidopsis* caused an increase in freezing tolerance, while knockdown of *CBF1* and/or *CBF3* decreases freezing tolerance after cold acclimation (Novillo et al., 2008). Previous studies have demonstrated that plant hormones play a crucial role in the cold stress response. For instance, ethylene-insensitive 3(*EIN3*), one of the transcription factors in the ethylene signaling pathway, restrains the *CBF* transcription, consequently suppressing the expression of downstream *COR* genes under normal growth conditions (Shi et al., 2012). At early stages of cold stress, a decrease in endogenous ethylene weakens the transcriptional inhibition of *CBFs* by ethylene signaling and triggers *CBF*-dependent cold acclimation. Subsequently, the cold stress response promotes the accumulation of EIN3 to prevent overaccumulation of *CBFs* through feedback adjustment (Shi et al., 2015). Furthermore, the accumulation of SA can also upregulate the expression of *COR* genes and improve the chilling tolerance of plants. Dong et al. (2014) demonstrated that chilling stress increased the expression levels of *CBF* and *COR47* in cucumber seedlings, and the increases in the expression of *CBF* and *COR47* were higher in SA-pretreated seedlings and lower in paclobutrazol (PAC, an inhibitor of SA biosynthesis)-pretreated seedlings than in control seedlings. However, how SA and COR genes influence stress responses under chilling stress remains unclear.

Grafting has been widely used to improve the tolerance of a plant to pathogens and to abiotic stresses, namely, low temperature, high temperature, drought, or salts, in the horticulture industry (Zhou et al., 2007; Han et al., 2013; Xing et al., 2015). Many rootstocks with strong ecological adaptability possess tolerance to exogenous limiting factors, such as pathogens, salinity, water, temperature, and oxygen deficit (Rizk-Alla et al., 2011; Marguerit et al., 2012; Li et al., 2014a). This resistance is mainly from the signal transduction between rootstocks and scions. Some studies suggest that rootstocks confer positive effects to the scion by alterations in chemical or root-to-shoot hydraulic signals (Li et al., 2014a). However, the interaction between rootstock and scion in promoting the tolerance of a plant to abiotic stress is still ambiguous. Dat et al. (1998) found that high temperature rapidly increased total endogenous SA. SA has been recognized as a long-distance hormone signaling molecule that is involved in the regulation of stress responses of plant and has been proven to be capable of regulating the responses of plant to heat stress and alleviating certain consequences induced by oxidative stress (Dat et al., 1998; Liu et al., 2003). However, the response of endogenous SA in rootstock/scion to chilling stress and its relationship with chilling tolerance in grafted plants remain elusive. Therefore, we compared the stress tolerance of cucumber and pumpkin plants grafted with their own roots to those grafted with pumpkin and cucumber roots, respectively, when exposed to root-zone chilling, aerial chilling, and the combination of the two. The results of this study suggest that pumpkin-induced SA accumulation upregulated the expression of *COR* genes and improved the chilling tolerance in cucumber, implicating that SA is involved in the rootstock–scion communication in responding to chilling stress in grafted cucumber.

## Materials and Methods

### Plant Materials and Growth Environment

Two different species, cucumber [*Cucumis sativus* L, cv. “Jinyou 35" (JY35), *Cs*] and pumpkin [*Cucurbita moschata* D., cv. Jinmama 519 (JM519), *Cm*], were used as experimental materials that serve as scion or rootstock for each other. The *Cs* and *Cm* seeds were soaked in water for 6 or 12 h, respectively, and germinated on a moist filter paper in the dark at 28°C for 24 or 36 h, respectively. For rootstocks, the germinated *Cm* seeds were sown into trays filled with seedling substrate, and for scion, *Cs* seeds were sown 3 days later. When the cotyledon of the scions expanded, the seedlings were grafted with the top approach grafting method. When the *Cs* was used as the rootstock, *Cm* seeds were sown 10 days later and then grafted when the first true leaf of rootstocks spread. The grafted combinations (scion/rootstock) were designated as *Cs/Cm*, *Cs/Cs*, *Cm/Cs*, and *Cm/Cm* and were grown in an artificial climate chamber with a constant humidity of 80–90% and a photon flux density (PFD) of 50 μmol m^−2^ s^−1^ and 25–28°C for 7 days. When the first true leaves fully expanded, the four grafted combination seedlings were transplanted into a container (40 cm × 25 cm × 15 cm) filled with 1/2 Hoagland’s nutrient solution and under the following environmental conditions: photoperiod of 11 h, PFD of 600 μmol m^−2^ s^−1^, temperature of 26/18°C (day/night), and relative humidity of 80%.

### Chilling and Salicylic Acid Treatment

To analyze the response of the grafted plants to aerial and/or root-zone chilling temperature, the three-leaf stage seedlings of the four grafted combinations were transferred to a climate chamber at low (5°C) or normal (25°C) temperature with a photoperiod of 11 h and PFD of 100 μmol m^−2^ s^−1^. For the shoot chilling stress treatment (5/25°C, shoot/root temperature), half of the nutrient solution of 5°C-treated seedlings in the climate chamber was heated to and maintained at 25°C with an external constant temperature circulator (HX-2015, Zhengzhou Great Wall, China), and the other half of the nutrient solution of seedlings was used as the combined chilling treatment (5/5°C). For the root-zone chilling stress treatment (25/5°C), half of the nutrient solution of 25°C-treated seedlings in the climate chamber was cooled to and maintained at 5°C, and the other half of the nutrient solution of seedlings was used as the control (25/25°C). During 48 h of chilling stress, leaf and root samples were collected to measure the electrolyte leakage (EL), malondialdehyde (MDA), photosynthetic rate (Pn), chlorophyll fluorescence parameters, mRNA abundances of *RCA* and *COR* genes, and the relative expression of *CBF1* at the protein level. The SA emission system was also measured after chilling stress for 120 h. All the experiments were repeated four times per treatment. For transcriptome analysis, the three-leaf stage *Cs/Cs* and *Cs/Cm* seedlings were exposed to 5 or 25°C (control) for 12 h. The second true fully expanded leaves were sampled for RNA-Seq, and each treatment had three biological replicates with 10 seedlings per replicate.

To determine the effect of SA on the chilling tolerance of grafted cucumber seedlings, *Cs/Cs* and *Cs/Cm* seedlings at the three-leaf stage were foliar sprayed with 1.0 mM SA, 0.03 mM 2-aminoindan-2-phosphonic acid (AIP, a synthesis inhibitor of SA; Solecka and Kacperska, 2003), 0.1 mM L-a-aminooxy-b-phenylpropionic acid (AOPP, a synthesis inhibitor of SA; Janas, 1993), or deionized water (control) 24 h before the combined chilling (5/5°C) treatment. After exposure to chilling stress for 48 h, the Pn and chlorophyll fluorescence parameters were determined, and leaf samples were collected immediately to assay the EL, MDA, chilling injury index (CI), and the relative expression of the *COR* genes at the mRNA and protein levels. The deionized water treatment under chilling stress was marked as the H_2_O treatment to distinguish the control under normal conditions. There were four replicates per treatment and 10–20 seedlings per replicate.

### Determination of CI, EL, and MDA Content

For CI determination, the stress seedlings were graded according to the method described by Semeniuk et al. (1986), and the CI was calculated as follows: CI = Σ (plants of different grade × grade)/[total plants × 5 (the maximum grade)]. The EL was assayed using the method of Campos et al. (2003). A total of 0.3 g of each sample was oscillated at 25°C in 25 ml test tubes containing 20 ml deionized water for 3 h, and the electrical conductivity (EC_1_) of the bathing solution was measured, with a conductivity meter (DDB-303A, Rex Electric Chemical, China). Then, the leaf samples were boiled (100°C) for 30 min, and the EC_2_ of the bathing solution was measured after cooling to room temperature. The EL was calculated as follows: EL (%) = EC_1_/EC_2_ × 100. The MDA content was determined as described by Zhao and Cang (2015), 0.5 g fresh tissue was extracted with 4 ml 10% trichloroacetic acid, and the homogenate was centrifuged at 4,000 *g* for 10 min at 4°C. Then, the supernatant was mixed with an equal volume of 0.6% thiobarbituric acid, added to water, and boiled for 15 min, and the absorbance of supernatant was measured at 450, 532 and 600 nm. The MDA content was calculated from the following equation: MDA (μmol L^−1^) = 6.45 × (OD_532_–OD_600_)−0.56 × OD_450_ to eliminate the effects of soluble sugar.

### Photosynthetic Rate and Chlorophyll Fluorescence Assay

The photosynthetic rate (*Pn*) was measured with a portable photosynthesis system (Ciras-3, PP-Systems International, Hitchin, Hertfordshire, United Kingdom). During the measurements, constant photon flux density (PFD, 600 μmol m^−2^ s^−1^), CO_2_ concentration (400 mg L^−1^), and leaf temperature (25 ± 1°C) were maintained.

The maximum photochemical efficiency of PSII in darkness (*F*_v_/*F*_m_) and actual photochemical efficiency (*Φ*_PSII_) were measured using a modulated chlorophyll fluorescence spectrometer (FMS-2, Hansatech Instruments, King’s Lynn, Norfolk, United Kingdom) according to the instructions. The chlorophyll fluorescence imaging was also visualized with a variable chlorophyll fluorescence imaging system (Imaging PAM, Walz, Wurzburg, Germany), which consist of a CCD camera, LED lights, and controlling unit connected to a PC running a dedicated software (Imaging Win 2.3, Walz, Wurzburg, Germany), by the method of Tian et al. (2017).

### Measurement of Salicylic Acid Content

Salicylic acid content was measured with a high-performance liquid chromatography-triple quadrupole mass spectrometry (HPLC-MS, Thermo Fisher Scientific, TSQ Quantum Access, USA) as described by Li et al. (2014b). About 0.2 g freeze-dried leaf or root was grounded and extracted with 5 ml of 80% methanol (containing 30 μg ml^−1^ sodium diethyldithiocarbamate) for 16 h at 4°C in darkness and then centrifuged at 8,000 *g* for 10 min at 4°C. The supernatant was collected, and the remaining residue was extracted two more times with the same method, each time for 2 h. Then, the homogenates were centrifuged at 8,000 *g* for 10 min at 4°C, and the supernatants were dried *in vacuo* at 38°C with a rotary evaporator (N-1210B, Shanghai EYELA, China). The resulted residues were dissolved in 5 ml of 0.2 M phosphate buffer (pH = 7.8) and 4 ml of trichloromethane. Let it standing for 30 min after oscillation, the solution was kept until stratification and the lower layer was discarded to remove the pigment. The phenolic impurities was removed by polyvinylpolypyrrolidone (PVPP), followed by centrifugation at 8,000 g for 10 min. The supernatants were adjusted to pH 3.0 with formic acid and were leached by ethyl acetate three times. The total ester phase was dried *in vacuo* at 38°C with a rotary evaporator. Finally, the residue was dissolved in 1.0 ml mobile phase (methanol: 0.04% acetic acid = 50:50, v:v) and filtered through a 0.22 μm filter. The filtrate was used for the HPLC-MS analysis.

Methanol and 0.04% acetic acid were used as mobile phases, at a column temperature of 30°C and a flow of 0.3 ml min^−1^ to separate SA with a Hypersil Gold C_18_ column (100 mm × 2.1 mm, 1.9 μm, Thermo Fisher Scientific, United States). The components separated by HPLC were further quantitatively analyzed by MS with triple quadrupole mass spectrometry with electrospray ionization (ESI) in negative mode. The optimum conditions for MS were ion source capillary temperature of 300°C, vaporizer temperature of 300°C, spray voltage of 2.5 kV, sheath gas pressure of 33 Arb, auxiliary gas pressure of 2 Arb, and the mode of selective response monitoring (SRM) adopted for scanning (Table 1). The SA standard sample (Sigma-Aldrich, United States) was used to generate the standard curve, and the SA content in plants was calculated by the external standard method.

**Table 1:** Characteristic fragment ions of the salicylic acid (SA) obtained by liquid chromatography-mass spectrometry (LC-MS) in selective response monitoring (SRM) mode.

Analyte	ESI mode	Transition 1		Transition 2	
		Quantitative ion	Collision energy/eV	Qualitative ion	Collision energy/eV
Salicylic acid	ESI-	137.055 > 93.137	17	137.055 > 65.268	36

### Activity of Salicylic Acid Synthase Enzymes Assay

The phenylalanine ammonia-lyase (PAL) activity was determined using a spectrophotometer (UV-2450, Shimadzu, Japan) as described by Shang et al. (2012). About 0.5 g fresh sample was extracted with 5 ml of 50 mM Tris–HCl buffer [containing 5 mM EDTA, 15 mM β-mercaptoethanol, and 0.15%(w/v) polyvinylpyrrolidone, pH 8.5]. The supernatant was reacted with 0.02 M phenylalanine at 37°C for 1 h. The amount of the enzyme required for the changes in absorbance of 0.01 h^−1^ at 290 nm is one unit (which equates to 1 μg trans-cinnamic acid was formed per ml reaction mixture).

### Transcriptome Analysis

Total RNA was extracted with Trizol reagent (Invitrogen, Carlsbad, CA, United States) and subsequently used for mRNA purification and library construction using the NanoDrop 2000 spectrophotometer (Thermo Scientific, United States) and TruSeq Stranded mRNA LT Sample Prep Kit (Illumina, San Diego, CA, United States), respectively, according to the instructions. Samples were sequenced on an Illumina NovaSeq 6000 (Illumina, San Diego, CA, United States), and the clean reads were mapped to the cucumber genome using HISAT2. Fragments per kilobase per million mapped reads (FPKM) of each gene were calculated using Cufflinks, and the read counts of each gene were obtained by HTSeq-count. We standardized the counts of genes in each sample and calculated the difference multiplied with DESeq (2012) R package. The value of *p* each gene was calculated, and the value of *p* was corrected by multiple hypotheses testing using the false discovery rate (FDR) error control method. We set *p* < 0.05 and fold change > 2 as the threshold for significant differential expression. The Kyoto Encyclopedia of Genes and Genomes (KEGG) pathway enrichment analysis of differentially expressed genes (DEGs) was performed, respectively, with R based on the hypergeometric distribution. This work was supported by Qingdao Oebiotech Co. Ltd (Qingdao, China).

### RNA Extraction and Gene Expression Analysis

Total RNA was extracted from cucumber leaves and roots using an RNA extraction kit (TRIzol; TRANs, Beijing, China) and then reverse-transcribed with HiScript® III RT SuperMix for qPCR (Vazyme, Nanjing, China). The relative expression of *RCA*, SA synthase genes, and *COR* genes in cucumber or pumpkin plants was analyzed by real-time quantitative PCR (RT-qPCR) using ChamQ™ Universal SYBR® qPCR Master Mix (Vazyme, Nanjing, China), according to the instructions. Amplification was performed on the LightCycler® 480II system (Roche, Penzberg, Germany). The primers for different genes are shown in Table 2.

**Table 2:** Primer sequences.

Gene	Accession number	Primer sequences
*CsActin*	XM_011659465	5'-AGAAGATCTGGCATCACA-3'
		5'-TCCAATCCAGACACTGTACT-3'
*CmActin*	XM_023107141	5'-CACGAAACTACCTACAACTCC-3'
		5'-CTCATCCTGTCAGCAATAC-3'
*CsICE1*	XM_011653285	5'-CGCATCGAGTTGGCTCTGGTG-3'
		5'-GTCCTCATCGCCGTTCATCTTCC-3'
*CsCBF1*	XM_004140746	5'-TACAGAGGAGTCAGGAGGA-3'
		5'-AGAATCGGCGAAATTGA-3'
*CsCOR47*	XM_011659051	5'-ACTTTGAGAGGACATTTGATG-3'
		5'-GAAGCTCCAATTTTGACTTG-3'
*CmICE1*	XM_023078346	5'-CTCCTCCTCCTCCTGGTTCTTCG-3'
		5'-GATTCGGCAGCATCGGTATCGG-3'
*CmCBF1*	XM_023067207	5'-TTCGAGGTCGCTCTGCTTGTC-3'
		5'-CCGCCGCCTTCTGAATATCC-3'
*CmCOR47*	XM_023090727	5'-TGTTCAAGAGGGTGGTGTCG-3'
		5'-GGATCGGGTGAGTTTCTCCA-3'
*CsRCA*	FJ980456	5'-AAAGTGGGCTGTAGGCGTTG-3'
		5'-TTTTCTATTGTCATCTTCGGTTGG-3'
*CmRCA*	XM_023099578	5'-TCTTGGTGGCACTACTCAATAC-3'
		5'-CATACCAGGAGCTGAACATT-3'
*PAL-CsaV3_6G039670*	XM_031887954	5'-ACTACTCATCCAGACCCACTCA-3'
		5'-GAGTAGCAATAGCAGCCACCT-3'
*PAL-CsaV3_6G039680*	XM_011659349	5'-TGTGCCATACCCATAGTGATCC-3'
		5′-AACACCGACTCTAGCGGACT-3'
*PAL-CsaV3_6G039720*	XM_004143212	5'-TGGCCCTAGTCGAGACCATT-3'
		5'-AGCGATAGCAGCAACTTGAGA-3'
*ICS-CsaV3_1G002150*	XM_011661668	5'-GCGTCGGAGGAAGTCAAGAA-3'
		5'-GGATTGGAACTTGGAGCCGA-3'
*SAMT-CsaV3_6G046470*	NM_001280750	5'-GGGAAACACCAGTTATGCCA-3'
		5'-CTTTGGTGAAGCTTTTGGCGA-3'
*SAMT-CsaV3_6G046500*	XM_004149946	5'-TTTTTCAACGGAGTCGCTGG-3'
		5'-CAACCATTCGTCCACCCGTTA-3'
*SAMT-CsaV3_6G046520*	XM_011660193	5'-CTCTTTAATGGGGTGCCTGGT-3'
		5'-CCACCACACTCTTTGGGCTT-3'
*SABP2-CsaV3_2G014230*	XM_004153623	5'-CATCGGGTGACAGCGTTAGA-3'
		5'-GCATAGCGGCAACCACAAAA-3'

### Sodium Dodecyl Sulfate-Polyacrylamide Gel Electrophoresis and Immunoblot Analysis

Total protein was extracted and then separated with a 10% (w/w) sodium dodecyl sulfate-polyacrylamide gel electrophoresis (SDS-PAGE) gel, and the resulted proteins from the gel were blotted onto polyvinylidene difluoride (PVDF) membranes. The blot was blocked with 5% (w/w) skimmed milk for 2 h at room temperature and subsequently incubated overnight with the primary antibody at 4°C. Afterward, the blot was incubated with the antibody of horseradish peroxidase-conjugated anti-rabbit IgG (CWBio, Beijing, China) for 2 h. The immunoreaction was detected using the ECL Western Blot Kit (CW00495, CWBio, Beijing, China), and the ChemiDoc™ XRS imaging system (Bio-Rad Laboratories Inc., Hercules, CA, United States) was used to record the chemiluminescence on blots. The primary CBF1 antibody was ordered from GenScript Company (Nanjing, China).

### Availability of Supporting Data

The data discussed in this publication have been deposited in Gene Expression Omnibus of NCBI and can be accessed through Sequence Read Archive (SRA) accession no: PRJNA701131.^1^

### Statistical Analysis

The whole experiment was performed at least triplicate, and results are shown as means ±one SD. All data were analyzed statistically using DPS soft. Statistical analysis of the values was determined at *p* < 0.05, according to Duncan’s multiple range tests.

## Results

### Rootstock–Scion Interactions Influence Plant Chilling Tolerance

**Figure 1:**
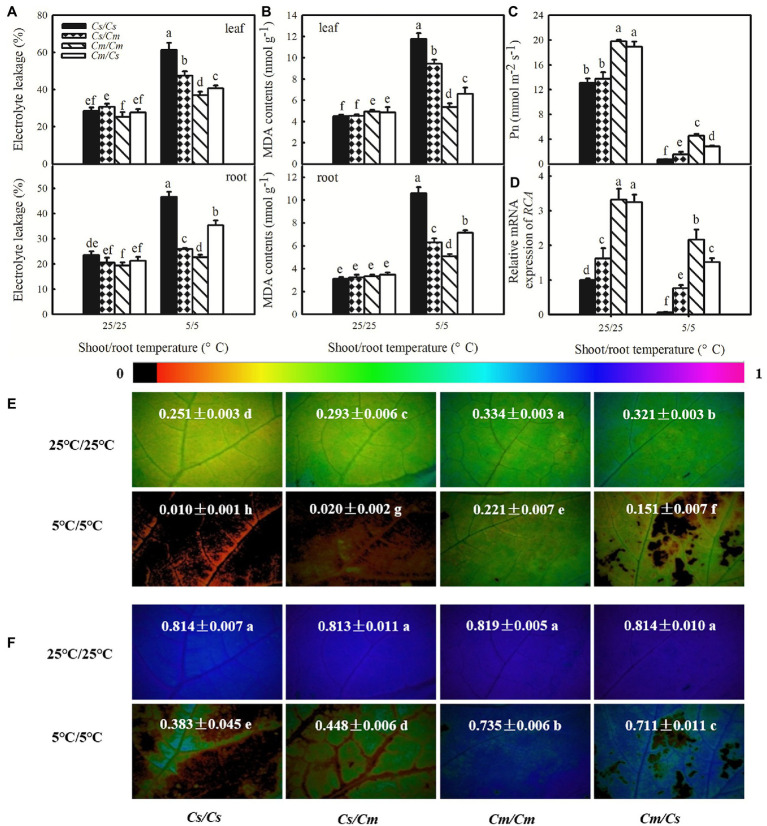
Effect of rootstock on the tolerance to chilling stress of own-root and hetero-root grafted cucumbers and pumpkins. **(A)** Electrolyte leakage (EL) of leaf and root; **(B)** malondialdehyde (MDA) content; **(C)** photosynthetic rate (Pn); **(D)**, relative expression of Rubisco activase (*RCA*); **(E,F)** images of actual photochemical efficiency (*Φ*_PSII_) and maximum photochemical efficiency (*F*_v_/*F*_m_)_,_ respectively, after exposure to 5°C for 48 h. The false color code depicted at the top of the image ranging from 0 (black) to 1.0 (purple) represents the degree of photoinhibition at PSII. Data are the means of four replicates (±SDs). Different letters indicate a significant difference between samples according to Duncan’s new multiple range test (*p* < 0.05).

To explore whether the rootstock–scion communication directly affects the chilling tolerance of grafted plants, we used the cold-tolerant species *Cm* and cold-sensitive species *Cs*, serving as scion or rootstock for each other and measured the changes in EL, MDA, Pn, *RCA* relative mRNA expression, *Φ*_PSII_, and *F*_v_/*F*_m_ in the grafted seedlings after exposure to aerial and/or root-zone chilling stress. At the optimum growth temperature (25/25°C), the own-root grafted (*Cs/Cs* and *Cm/Cm*) and hetero-root grafted plants (*Cs/Cm* and *Cm/Cs*) showed similar EL and MDA in both leaves and roots. Chilling stress (5/5°C) led to significant increases in the accumulation of EL and MDA in all plants (Figures 1A,B). Interestingly, the hetero-grafted cucumber plants that used *Cm* as rootstock (*Cs/Cm*) showed distinctly lower accumulation of EL and MDA in leaves, compared with the *Cs/Cs* plants. However, the hetero-grafted pumpkin plants using cucumber as rootstock (*Cm/Cs*) had higher EL and MDA in leaves than the *Cm/Cm* plants after chilling stress (*p* < 0.05). Moreover, the EL and MDA in *Cs/Cm* roots were significantly lower than those in *Cs/Cs* roots, but those in the *Cm/Cs* roots were markedly higher than in *Cm/Cm* roots (*p* < 0.05).

The Pn and mRNA abundance of *RCA* decreased under chilling stress in all the own-root and hetero-root grafted plants, but the extent of the decrease varied (Figures 1C,D). After 48 h at 5/5°C, the Pn in *Cs/Cs* plants decreased by 94.1% but that in *Cs/Cm* plants decreased by 88.9%. In contrast, the Pn in *Cm/Cm* plants only decreased by 77.1% after exposure to 5/5°C for 48 h, while that in *Cm/Cs* plants decreased by 85.0%. The *Cs/Cm* plants showed significantly higher *RCA* mRNA abundance than the *Cs/Cs* plants following 48 h chilling stress, but the *Cm/Cs* plants showed lower mRNA abundance of *RCA* relative expression to the *Cm/Cm* plants (*p* < 0.05).

Figure 1E reveals that chilling stress also caused significant decreases in *Φ*_PSII_ of the own-root and hetero-root grafted plants. The decrease in *Φ*_PSII_ in *Cs/Cm* plants was less than that in *Cs/Cs* plants, but decrease in *Φ*_PSII_ in *Cm/Cs* plants was markedly greater than that in *Cm/Cm* plants (*p* < 0.05). Plants subjected to 5/5°C stress revealed a remarkably lower *F*_v_/*F*_m_ than the 25/25°C control plants (Figure 1F). After exposure to 5/5°C for 48 h, the *F*_v_/*F*_m_ in *Cs/Cm* plants was 18.4% higher than that in *Cs/Cs* plants but that in *Cm/Cs* plants was 8.2% lower than that in *Cm/Cm* plants. These data suggest that grafting with chilling-tolerant pumpkin as rootstock can enhance the tolerance of cucumber to chilling stress while that with cold-sensitive cucumber as rootstock may decrease the chilling tolerance of pumpkin.

### Pumpkin (*Cm*) Rootstock Upregulated the Expression of *COR* Genes in Grafted Plants

**Figure 2:**
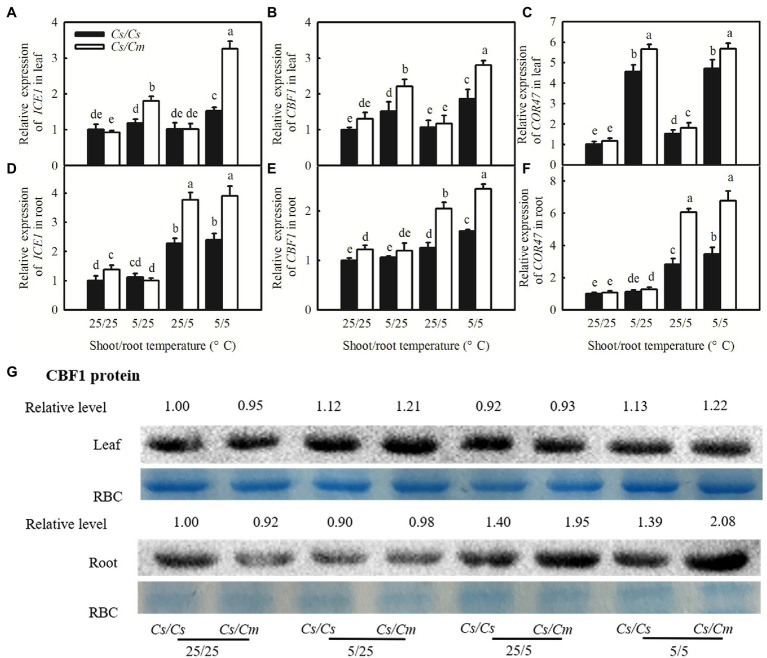
Changes of the relative mRNA expression of C-repeat-binding factor (*CBF1*), inducer of *CBF* expression (*ICE1*), cold-responsive (*COR47*) genes, and the protein level of *CBF1* in the *Cs/Cs* and *Cs/Cm* plants under aerial or/and root-zone chilling stress. **(A–F)** Relative mRNA expression of *ICE1*, *CBF1*, and *COR47* in leaves and roots; **(G)** the protein level of *CBF1* in leaf and root. Three-leaf stage seedlings were exposed to chilling stress for 48 h. Data are the means of four replicates (±SDs). Different letters indicate a significant difference between samples according to Duncan’s new multiple range test (*p* < 0.05).

The ICE1–CBF–COR transcriptional cascade is a key cold acclimation signaling pathway. To examine whether the transcriptional cascade contributed to *Cm*-induced chilling tolerance in grafted cucumber plants and *Cs*-induced chilling sensitivity in grafted pumpkin plants, we compared the responses of the C-repeat-binding factor (*CBF1*), an inducer of *CBF* expression (*ICE1*), and *COR47* genes to chilling stress among the four grafted combinations of *Cs/Cs*, *Cs/Cm*, *Cm/Cm*, and *Cm/Cs*. Figures 2A–C shows that 5/25 and 5/5°C stress largely upregulated the relative mRNA expression of *ICE1*, *CBF1*, and *COR47* in the leaves of grafted plants (*p* < 0.05), but 25/5°C did not affect them. The increases in the relative mRNA expression of the three genes were markedly higher in *Cs/Cm* than in the *Cs/Cs* leaves. For example, the mRNA abundance of *CBF1* increased by 69.6 and 101.5% in *Cs/Cm* leaves but only by 51.8 and 86.1% in *Cs/Cs* leaves, after exposure to 5/25 and 5/5°C for 48 h, respectively.

To further explore whether the increased relative mRNA expression of *ICE1*, *CBF1*, and *COR47* in *Cs/Cm* leaves was transported from roots or whether those in *Cm/Cs* roots was transported from leaves, we determined the relative mRNA expression of *ICE1*, *CBF1*, and *COR47* in roots of the four grafted combinations. The mRNA abundances of *ICE1*, *CBF1*, and *COR47* increased only in the roots of 25/5°C- and 5/5°C-stressed plants, but similar *ICE1*, *CBF1*, and *COR47* mRNA abundances were found in the roots of 5/25°C-stressed or control plants. *Cs/Cm* plants showed significantly higher mRNA abundances of *ICE1*, *CBF1*, and *COR47* in roots than *Cs/Cs* plants (Figures 2D–F). Aerial or aerial and root-zone chilling also significantly increased the accumulation of *CBF1* protein in leaves, which were more evident in *Cs/Cm* leaves than in *Cs/Cs* leaves (Figure 2G). *CBF1* protein in roots of the four grafted combinations was significantly upregulated at 25/5 and 5/5°C but not induced at 5/25°C. The increase in the *CBF1* protein was greater in *Cs/Cm* than in *Cs/Cs* roots under 25/5 and 5/5°C chilling stress. These results indicate that the increase in *CBF1* protein in *Cs/Cm* leaves was mainly synthesized in leaves but not transported from roots. Interestingly, the increase in the mRNA and protein levels of *COR* genes was less in *Cm/Cs* leaves than in *Cm/Cm* leaves under 5/25 and 5/5°C stress. *Cm/Cs* roots also displayed lower mRNA abundances of *ICE1*, *CBF1*, and *COR47*, and lower protein level of *CBF1* compared with the *Cm/Cm* roots at 25/5 and 5/5°C (Supplementary Figure 1).

### Transcriptome Analysis of the Own-Root and Hetero-Root Grafted Cucumber Leaves

**Figure 3:**
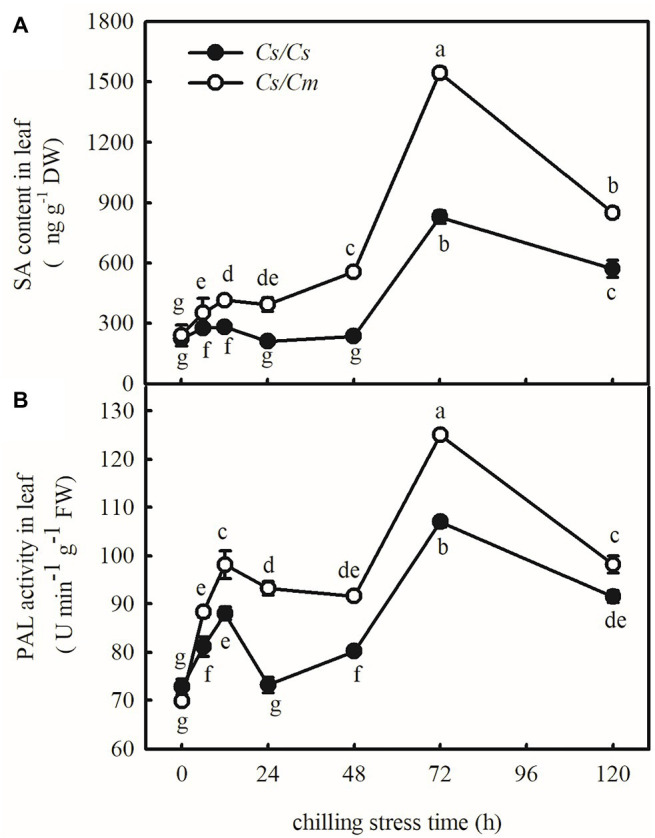
Response of salicylic acid (SA) accumulation and phenylalanine ammonia-lyase (PAL) activities to chilling stress in *Cs/Cs* and *Cs/Cm* plants. **(A)** SA content and **(B)** PAL activity. Three-leaf stage seedlings were exposed to 5°C for 120 h and sampled at 0, 6, 12, 24, 48, 72, and 120 h. Data are the means of four replicates (±SDs). Different letters indicate a significant difference between samples according to Duncan’s new multiple range test (*p* < 0.05).

To explore the response of endogenous SA to chilling stress, we estimated the endogenous SA accumulation and PAL activity in *Cs/Cs* and *Cs/Cm* plants under chilling stress for 120 h. Chilling stress caused a clear increase in the SA content and PAL activity at the first 12 h but then decreased. Twenty-four hours later, the SA content and PAL activity gradually increased along with the chilling time, and the increases were the most significant after 72 h at 5°C. Subsequently, the SA content and the PAL activity slowly decreased, but those in *Cs/Cm* plants were always higher than those in *Cs/Cs* plants (*p* < 0.05, Figure 3).

**Figure 4:**
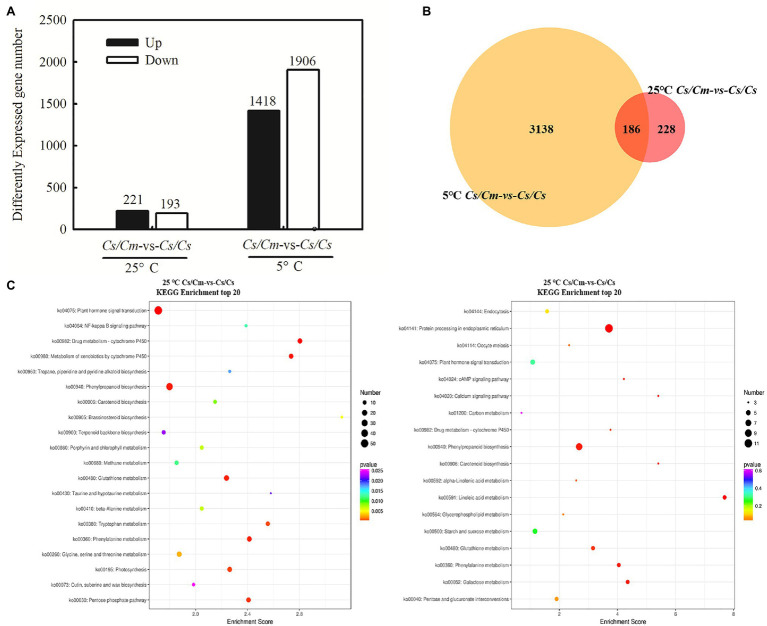
Comparison of gene expression and statistics of pathway enrichment. **(A)** Differentially expressed genes (DEGs) numbers (*p* ≤ 0.05 and fold change ≥2) between *Cs/Cm* and *Cs/Cs*; **(B)** Venn diagram; and **(C)** the DEG statistics of the Kyoto Encyclopedia of Genes and Genomes (KEGG) pathway enrichment on *Cs/Cm* vs. *Cs/Cs* under normal and chilling condition. The top 20 pathways with the most minimum values of *p* were displayed. The circle represents the gene number of the KEGG pathway. The circle color represents the genes enrichment degree of the KEGG pathway. Three-leaf stage seedlings were sampled at 0 or 12 h after chilling treatment.

To verify whether SA is involved in *Cm*-induced chilling tolerance in grafted cucumber, we analyzed the transcriptome in *Cs/Cs* and *Cs/Cm* plants. Compared with *Cs/Cs* plants, *Cs/Cm* plants upregulated 221 genes and downregulated 193 genes at the optimum growth temperature. After exposure to 5°C for 12 h, the *Cs/Cm* plants upregulated 1,418 genes and downregulated 1,906 genes relative to the *Cs/Cs* plants (Figure 4A), and there is an overlap of 186 differential genes between the two conditions of 25 and 5°C (Figure 4B). From the KEGG enrichment results (Figure 4C), we found that the obvious enrichment pathways (*p* < 0.05) of the DEGs in *Cs/Cm* plants were the hormone signaling pathways, phenylalanine metabolism, and phenylpropanoid biosynthesis relative to *Cs/Cs* plants under chilling stress, which were all closely related to SA synthesis and metabolism (Supplementary Figure 2).

**Figure 5:**
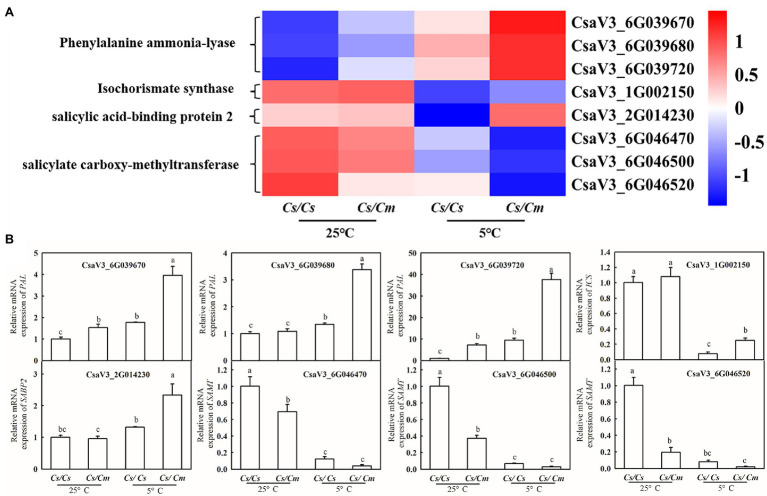
Verification of RNA-seq results using qRT-PCR. **(A)** Hierarchical clustering analysis of transcriptome data; **(B)** relative mRNA expression of *PAL*, *ICS*, *SAMT*, and *SABP2* in *Cs/Cm* and *Cs/Cs* leaves. Three-leaf stage seedlings were sampled at 12 h after chilling treatment. Data are the means of four replicates (±SDs). Different letters indicate a significant difference between samples according to Duncan’s new multiple range test (*p* < 0.05).

To further verify the results of transcriptome analysis, we chose some key related genes of SA synthesis and metabolism for qPCR assays. As shown in the heat maps (Figure 5A), the mRNA levels of *PAL*, *ICS*, and *SABP2* were upregulated, while *SAMT* was downregulated in *Cs/Cm* leaves, compared with *Cs/Cs* leaves, after chilling for 12 h. Then, we determined the relative mRNA expression of *PAL*, *ICS*, *SAMT*, and *SABP2* in *Cs/Cm* and *Cs/Cs* leaves at normal and chilling conditions. As shown in Figure 5B, the mRNA abundances of *PAL*, *ICS*, and *SABP2* were significantly higher in *Cs/Cm* leaves than in *Cs/Cs* leaves but that of *SAMT* was markedly lower in *Cs/Cm* leaves relative to the *Cs/Cs* leaves. These were highly consistent with the transcriptome data, which showed the confidence of the RNA-Seq data, and these results illustrate that SA is involved in Cm-induced chilling tolerance in hetero-root grafted cucumber.

### Rootstock–Scion Interaction Regulated Salicylic Acid Biosynthesis in Grafted Plants

**Figure 6:**
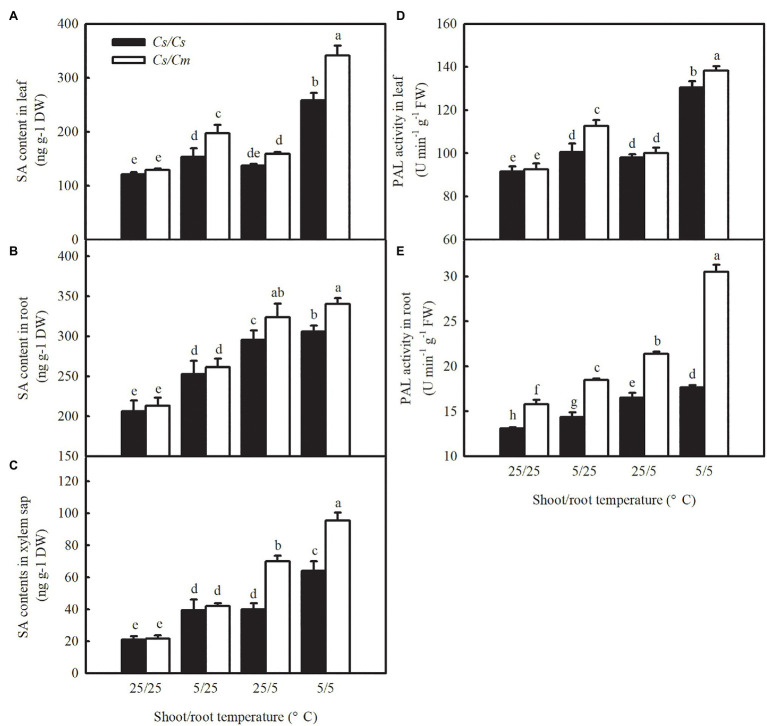
SA accumulation and PAL activity in leaves, roots, and xylem sap under aerial and/or chilling stress in *Cs/Cs* and *Cs/Cm* plants. **(A–C)** SA content in leaves, roots, and xylem sap, respectively; **(D,E)** PAL activity in leaves and roots, respectively. The third leaf of three-leaf stage seedlings was sampled at 12 h after chilling treatment. Data are the means of four replicates (±SDs). Different letters indicate a significant difference between samples according to Duncan’s new multiple range test (*p* < 0.05).

It is widely known that SA is an important signal involved in the responses of plants to biotic and abiotic tolerances. Therefore, we analyzed the changes in SA content in leaf, root, and xylem sap of the four grafted combinations under chilling stress. SA content and PAL activity increased remarkably (*p* < 0.05) in both leaves (Figures 6A,D) and roots (Figures 6B,E) of the grafted plants after exposure to 5/25, 25/5, and 5/5°C for 12 h, and the increase was significantly greater in the 5/5°C treatment. The rootstock genotypes did not affect the SA content in grafted plant leaves at 25/25°C, but the SA accumulations in *Cs/Cm* leaves increased by 52.5, 23.3, and 164.4% after 12 h at 5/25, 25/5, and 5/5°C, respectively, and were higher than those in *Cs/Cs* leaves. Notably, the increase in SA content in *Cs/Cm* leaves was accompanied by a significant increase in roots, but not in PAL activity in leaves, compared with *Cs/Cs* plants at 25/5°C. However, no differences were found in SA content in roots between *Cs/Cm* and *Cs/Cs* plants at 5/25°C, although the PAL activity increased significantly in *Cs/Cm* roots. Under combined chilling of the aerial and root zones, the increases in SA accumulation and PAL activity in *Cs/Cm* plants were significantly higher than those in *Cs/Cs* plants. To further explore whether there is a long-distance information transmission between rootstock and scion, we determined the changes in the SA content in xylem sap under aerial or root-zone chilling stress. Figure 6C showed that the SA content in the xylem sap increased in all of the grafted combinations after exposure to aerial, root-zone, and combined chilling stress. The increase in SA content in xylem sap was much higher in the *Cs/Cm* plants than in *Cs/Cs* plants after exposure to 25/5 and 5/5°C, but no significant differences were found in it between *Cs/Cm* and *Cs/Cs* at 5/25°C. These data suggest that SA acts as a long-distance signal and is involved in graft-induced chilling tolerance in cucumber plants. The increase in SA accumulation in *Cs/Cm* leaves is mainly attributed to the SA biosynthesis in leaves under aerial chilling stress, whereas at root-zone chilling conditions, the increase in SA content in *Cs/Cm* leaves is derived from the promotion of SA biosynthesis in roots.

From Supplementary Figure 3, we found that the SA content in *Cm/Cs* roots was significantly lower than that in *Cm/Cm* roots after 12 h at 5/25, 25/5, and 5/5°C. The decrease in SA content in *Cm/Cs* roots was accompanied by an obvious decrease in leaves but not in PAL activity in leaves in comparison with *Cm/Cm* under 25/5°C. The SA accumulation in the xylem sap was lower in the *Cm/Cs* plants than in *Cm/Cm* plants after exposure to 25/5 and 5/5°C, while no difference in it was found between *Cm/Cs* and *Cm/Cm* plants at 25/25 or 5/25°C. These results further confirmed that SA has been involved in rootstock–scion communication and plays a positive role in graft-induced chilling tolerance in plants.

### Salicylic Acid- or *Cm* Rootstock-Induced Expression of *COR* Genes and Chilling Tolerance in Cucumber

**Figure 7:**
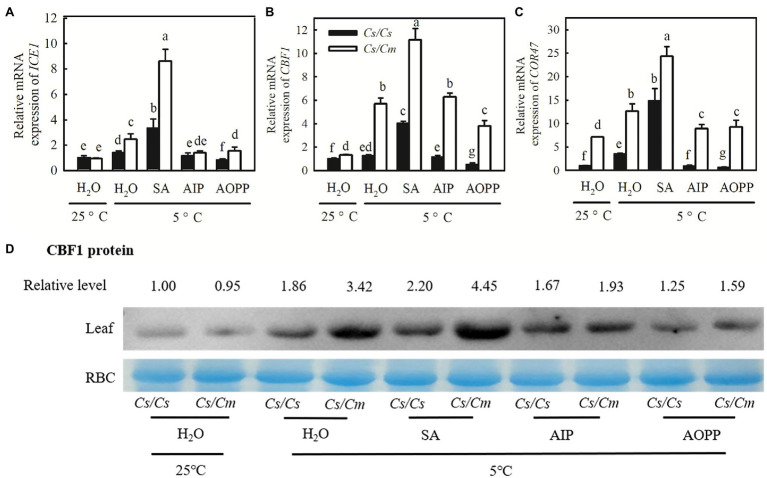
Effect of SA on the mRNA abundance of cold-responsive (*COR*) genes and the protein level of *CBF1* in *Cs/Cm* and *Cs/Cs* plants under chilling stress. **(A–C)** Relative mRNA expression of *ICE1*, *CBF1*, and *COR47*. Plants were foliar sprayed with 1.0 mM SA, 0.03 mM AIP, 0.1 mM AOPP, or deionized water (H_2_O, control) for 24 h and then exposed to 5°C stress for 48 h. **(D)** *CBF1* protein accumulation in seedlings after exposure to 5°C for 24 h. Data are the means of four replicates (±SDs). Different letters indicate significant a difference between samples according to Duncan’s new multiple range test (*p* < 0.05).

The real-time quantitative (qRT)-PCR results revealed that SA significantly upregulated the mRNA abundances of *ICE1*, *CBF1*, and *COR47* under chilling stress, and the increase in the mRNA abundances of the three genes was much greater in *Cs/Cm* than in *Cs/Cs* plants (Figures 7A–C). After chilling for 48 h, the transcript expression of *ICE1*, *CBF1*, and *COR47* in SA-pretreated *Cs/Cs* plants was upregulated by 2.3-, 3.1-, and 13.8-fold, respectively, and those in SA-pretreated *Cs/Cm* plants were increased by 7.6-, 10.1-, and 23.3-fold, respectively. However, plants treated with AIP and AOPP showed little difference in the relative mRNA expression of *ICE1*, *CBF1*, and *COR47* compared with the plants treated with H_2_O under chilling stress. Similar results were found at the protein level of *CBF1*, with stronger protein signals in SA-pretreated plants and weaker signals in AIP- and AOPP-pretreated plants than in H_2_O-treated plants under chilling stress (Figure 7D).

**Figure 8:**
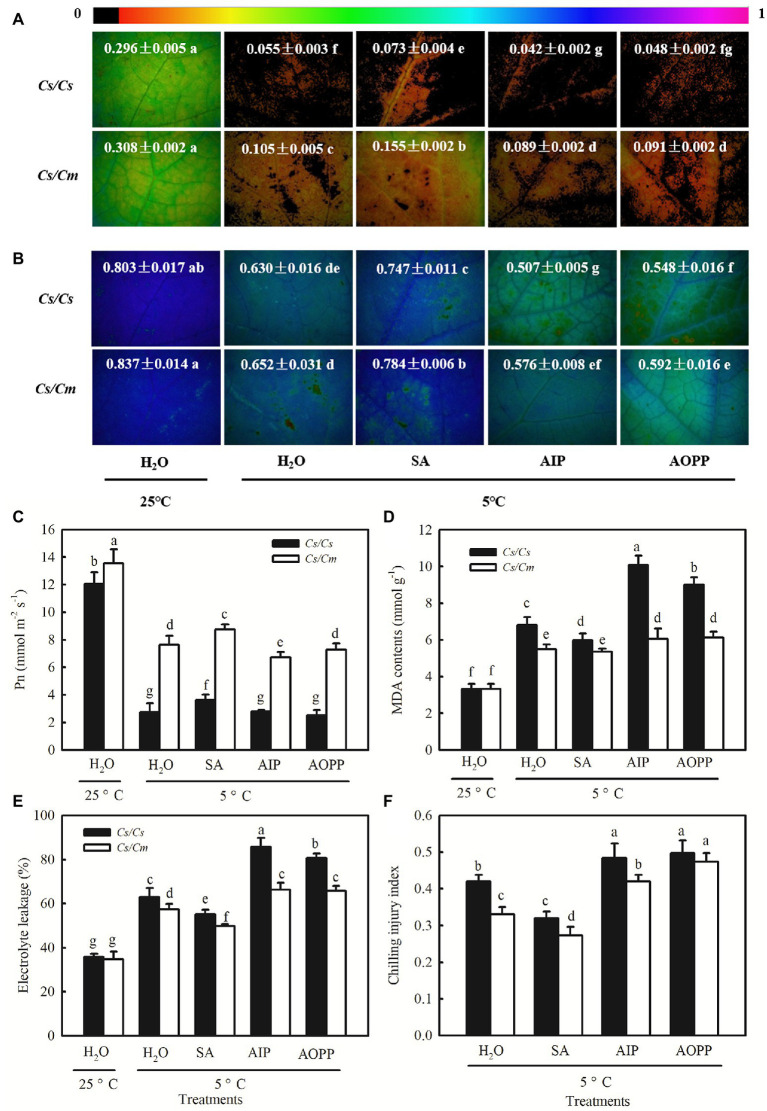
Effect of SA on the chilling tolerance of *Cs/Cm* and *Cs/Cs* plants. **(A)** *Φ*_PSII_; **(B)** *F*_v_/*F*_m_; **(C)** Pn; **(D)** MDA content; **(E)** EL; and **(F)** chilling injury index (CI). Plants were foliar sprayed with 1.0 mM SA, 0.03 mM AIP, 0.1 mM AOPP, or deionized water (control) for 24 h and then exposed to 5°C for 48 h. Data are the means of four replicates (±SDs). Different letters indicate a significant difference between samples according to Duncan’s new multiple range test (*p* < 0.05).

Chilling stress led to a significant decrease in *Φ*_PSII_ and *F*_v_/*F*_m_ in cucumber plants (Figures 8A,B). Notably, the chilling-induced decreases in *Φ*_PSII_ and *F*_v_/*F*_m_ were greatly reduced by SA but enhanced by AIP and AOPP (*p* < 0.05). The *Cs/Cm* plants showed higher *Φ*_PSII_ and *F*_v_/*F*_m_ than the *Cs/Cs* plants under chilling stress (*p* < 0.05). When the plants were subjected to chilling stress for 48 h, the Pn in H_2_O-treated *Cs/Cm* and *Cs/Cs* plants was decreased by 43.7 and 77.3%, respectively, but that in SA-treated *Cs/Cm* and *Cs/Cs* plants decreased by only 35.6 and 69.9%, respectively, relative to the control. AIP and AOPP treatments showed little differences in Pn compared with the H_2_O-treated *Cs/Cm* and *Cs/Cs* plants under chilling stress (Figure 8C). The chilling-induced accumulation of MDA and the EL and CI were reduced by SA but enhanced by AIP and AOPP (*p* < 0.05). At 8/5°C for 48 h, the *Cs/Cm* plants revealed significantly lower MDA, EL, and CI than the *Cs/Cs* plants (Figures 8D–F). These data suggest that exogenous SA significantly upregulated the expression level of *COR* genes in grafted cucumber, and it is involved in *Cm* rootstock-induced accumulation of the COR protein and subsequent chilling tolerance.

## Discussion

Despite the widespread use of grafting in crops, the mechanism by which scion–rootstock interaction regulates the tolerance of plants to abiotic stress is yet to be completely understood. In this study, we confirmed that the SA biosynthesis-associated genes (*PAL*, *ICS*, and *SABP2*) were significantly upregulated in *Cs/Cm* plants relative to the *Cs/Cs* plants under chilling stress. The data presented here also provide evidence that the *Cm* rootstock increases SA biosynthesis in roots and leaves when the roots or shoots are exposed to chilling stress. The increased SA accumulation in *Cs/Cm* plants under chilling stress promoted the expression of *COR* genes at the mRNA and protein levels and enhanced the photosynthetic assimilation and subsequent chilling tolerance. These results suggest that SA-induced changes in the expression of *COR* genes play a critical role in rootstock–scion communication in response to aerial or root-zone chilling stress in grafted plants.

### Rootstock Altered the Chilling Tolerance and Expression of *COR* Genes in Grafted Plants

It is well known that grafting with stress-tolerant rootstocks can improve tolerance to abiotic stresses (Li et al., 2014a; Shibuya et al., 2015; Xing et al., 2015; Sabatino et al., 2018). On the other hand, there are fewer studies about the effects of shoot and root relationships on stress tolerance. Previous studies found that rootstocks influence the growth and development of scion in several ways, such as vegetative vigor, stress tolerance, yield, and quality, and affect the agricultural traits crops (Lee et al., 2010; Gregory et al., 2013). The effect of rootstock on scions may be attributed to the variation of the chemical signaling of root-to-shoot and/or shoot-to-root (Gregory et al., 2013). Several studies on long-distance signaling about graft union provide evidence for multiple types of mobile signals, such as hormones, proteins, ribonucleoproteins, RNAs, small RNAs, and minerals (Kumari et al., 2015). In this study, we found that using pumpkins with higher chilling tolerance as rootstock can enhance the tolerance of cucumber shoots to chilling stress, as evidenced by the lower levels of EL and MDA accumulation as well as the decrease in Pn, *RCA* relative mRNA expression, *Φ*_PSII_, and *F*_v_/*F*_m_ in *Cs/Cm* plants, compared with the *Cs/Cs* plants under chilling stress (Figure 1). In contrast, *Cm/Cs* plants showed much higher EL and MDA contents and significantly lower Pn, *RCA* relative mRNA expression, *Φ*_PSII_, and *F*_v_/*F*_m_ than *Cm/Cm* plants after chilling stress (*p* < 0.05), indicating that using cold-sensitive cucumber as rootstock can decrease the cold tolerance of pumpkin. This result suggests that there is an interaction between rootstock and scion and that rootstock–scion communication directly affects the chilling tolerance of grafted plants.

The known major cold signaling pathway in higher plants is the *CBF*-mediated transcriptional regulatory cascade (Shi et al., 2015). A growing number of evidence suggests that various hormones are involved in responses to chilling stress and regulate chilling tolerance by either *CBF*-dependent or *CBF*-independent pathways in plants (Shi et al., 2015). For example, Hu et al. (2013) found that repressors of jasmonate acid (JA) signaling, the basic helix–loop–helix (bHLH) interacting proteins JAZ1/4, can regulate *CBF* expression and chilling tolerance by interacting with ICE1/2 and repressing ICE1 transcriptional activity. We also found that *Cm*-induced chilling tolerance in *Cs*/*Cm* was accompanied by remarkable increases in the mRNA abundances of *ICE1*, *CBF1*, and *COR47*, and the protein level of *CBF1* in leaves and roots (Figure 2). In contrast, *Cs*-induced chilling sensitivity in *Cm*/*Cs* plants coupled with the decreases in the relative expression of *COR* genes in mRNA and protein levels in leaves and roots (Supplementary Figure 1) implies that the use of *Cm* as a rootstock may improve chilling tolerance by increasing the expression of *ICE1*, *CBF1*, and *COR47* in hetero-grafted cucumber plants, while the use of *Cs* as a rootstock can reduce chilling tolerance by decreasing the *COR* genes in hetero-grafted pumpkin plants.

### Salicylic Acid Is Involved in *Cm*-Induced Upregulation of *COR* Genes and Chilling Tolerance

Salicylic acid has been demonstrated to be an important signaling molecule for the adaptation of plants to abiotic stresses, such as salt, chilling, and heat (Pál et al., 2013; Miura and Tada, 2014; Janda and Ruelland, 2015). Application of SA significantly improved freezing or chilling tolerance as evidenced by decreased ion leakage and alleviated oxidative stress (Shin et al., 2018; Pan et al., 2020). The present data showed that *Cs/Cm* plants had higher SA accumulation than *Cs/Cs* plants during chilling stress (Figure 3). To further provide the evidence for the relationship between *Cm*-induced chilling tolerance and SA, we determined the change in transcriptome level in *Cs/Cm* and *Cs/Cs* leaves under chilling stress. As expected, the *Cs/Cm* plants upregulated 1,418 genes and downregulated 1,906 genes, which were mainly enriched in the SA-associated pathways, such as hormone signaling pathways, phenylalanine metabolism, and phenylpropanoid biosynthesis (*p* < 0.05), through KEGG analysis (Figure 4). The relative mRNA expression of *PAL*, *ICS*, and *SABP2* involved in SA biosynthesis was significantly upregulated while that of SA metabolism-associated gene, *SAMT*, was markedly downregulated in *Cs/Cm* leaves under chilling stress, which was the main reason for increased SA accumulation in leaves of *Cs/Cm* plants (Figure 5). These data indicate that SA signaling is involved in chilling tolerance in both cucumber and pumpkin and agrees with results from previous studies (Shin et al., 2018; Pan et al., 2020). In accordance with these observations, foliar applications of SA also improved the chilling tolerance of *Cs/Cm* and *Cs/Cs* plants (Figure 8).

Salicylic acid could upregulate the expression of chilling response genes (*ICE*, *CBF1*, and *COR*) under chilling stress (Pan et al., 2020). Dong et al. (2014) found that chilling-induced expression of *CBF* and *COR47* in cucumber was blocked by the SA synthetic inhibitor, PAC, but rescued by the exogenous application of SA. In the current study, we also found that 1 mM of exogenous SA increased the mRNA expression of *ICE1*, *CBF1*, and *COR47* (Figures 8A–C) and the protein level of *CBF1* (Figure 8D) under chilling stress, compared with H_2_O treatment. Primarily, chilling stress caused a further increase in SA-induced expression of *ICE1*, *CBF1*, and *COR47* in *Cs/Cm* and *Cm/Cm* plants relative to the *Cs/Cs* and *Cm/Cs* plants, respectively, suggesting that the upregulation of *COR* genes by SA may be a mechanism of *Cm*-induced chilling tolerance.

Salicylic acid exists universally in plants and accumulates, for example, in *Arabidopsis* root tips, wheat seeds, and grapefruits under chilling stress (Scott et al., 2004; Wan et al., 2009; Kosová et al., 2012). Liu et al. (2003) studied this phenomenon using radioactivity technology and observed that when some leaves of a grape plant were subjected to heat stress, SA in the untreated part was quickly transported to the heat-treated part and induced heat tolerance together with SA in the heat-treated part. In this study, we discovered that the cold-sensitive *Cs/Cs* plants showed lower SA biosynthesis in leaves when exposed to aerial chilling stress in comparison with the *Cs/Cm* plants (Figure 6). Notably, SA accumulation in leaves was also caused by root-zone chilling stress, which was mainly due to the increased SA biosynthesis in roots. Compared with the *Cs/Cs* plants, the *Cs/Cm* plants showed higher amounts of SA following aerial, root-zone, and combined chilling stress. In contrast, the *Cm/Cs* plants revealed lower SA accumulation after exposure to chilling stress relative to the *Cm/Cm* plants (Supplementary Figure 3). Interestingly, the increase in SA accumulation in *Cs/Cm* leaves was accompanied by a significant increase in SA in roots and xylem sap but with a small change in PAL activity in leaves under 25/5°C treatment. However, the accumulation of SA in *Cs/Cm* leaves at 5/25°C was accompanied by an increase in PAL activity in leaves, with no significant difference in SA content in roots or xylem sap between *Cs/Cs* and *Cs/Cm* plants. These results indicate that SA acts as a long-distance signal to improve chilling tolerance in *Cs/Cm* plants. The *Cm* rootstock increased the chilling tolerance of the hetero-grafted cucumber plants by increasing SA biosynthesis in leaves under aerial chilling stress through unknown signal(s) from the roots and by increasing SA biosynthesis in roots under root-zone chilling stress.

## Conclusion

**Figure 9:**
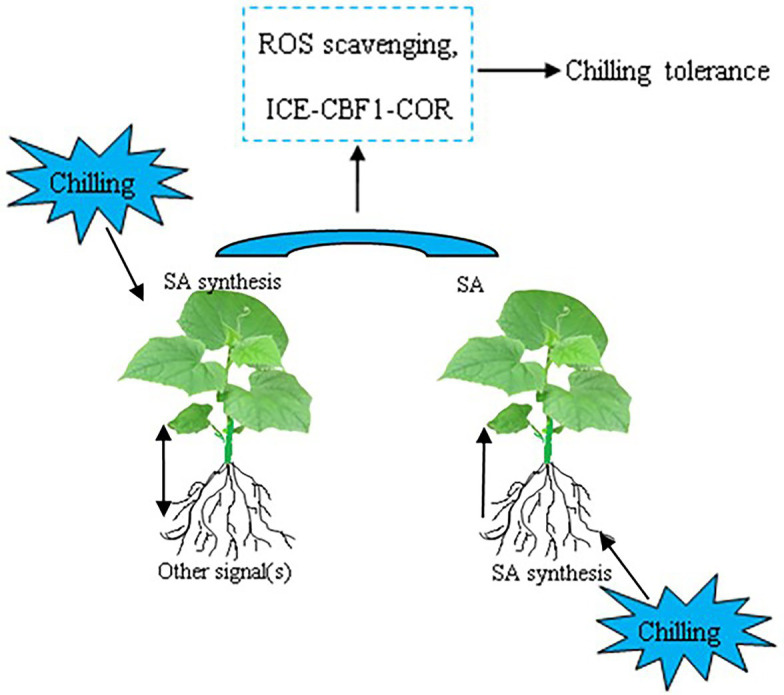
Simplified schematic model for SA-induced expression of COR genes and chilling tolerance in grafted cucumber plants responding to chilling stress. The original contributions presented in the study are publicly available. These data can be found here: Gene Expression Omnibus of NCBI, Sequence Read Archive (SRA) accession no: PRJNA701131 (http://www.ncbi.nlm.nih.gov/bioproject/701131).

In conclusion, the data presented here demonstrated that grafting with cold-tolerant *Cm* as rootstock enhances the chilling tolerance of hetero-grafted cucumber, while grafting with the cold-sensitive *Cs* as rootstock decreases the chilling tolerance of hetero-grafted pumpkin. Chilling tolerance is positively correlated with the accumulation of SA and the relative expression of COR genes in plants. The increase in SA accumulation in *Cs/Cm* leaves is attributed to an increase in SA biosynthesis in leaves and an increase in transportation from roots under aerial or root-zone chilling stress, respectively (Figure 9). The upregulation of the expression of COR genes with SA may be a mechanism of *Cm*-induced chilling tolerance. These findings have presented strong evidence that SA is involved in rootstock–scion communication under chilling stress and plays an important role in improving chilling tolerance in grafted cucumber. Further studies using advanced molecular techniques analyses are required to better explore the detailed mechanisms of rootstock–scion communication and the interactive role of SA and other signals in grafting-induced stress tolerance in plants.

## Data Availability Statement

The original contributions presented in the study are publicly available. This data can be found here: Gene Expression Omnibus of NCBI, SRA accession: PRJNA701131 (http://www.ncbi.nlm.nih.gov/bioproject/701131).

## Author Contributions

XF performed most of the experiments, analyzed the data, and completed the first draft. X-ZA and H-GB designed the research and edited the study. Y-QF, X-WZ, and Y-YZ worked together with XF to accomplish the experiment. All authors contributed to the article and approved the submitted version.

### Conflict of Interest

The authors declare that the research was conducted in the absence of any commercial or financial relationships that could be construed as a potential conflict of interest.

## Footnotes

^1^https://dataview.ncbi.nlm.nih.gov/object/PRJNA701131?reviewer=49vedjjg5erb2c7g2beru1ucb1
