# Supplementary material for: Salicylic Acid Is Involved in Rootstock–Scion Communication in Improving the Chilling Tolerance of Grafted Cucumber
Source: Front Plant Sci. 2021 Jun 24;12:693344. doi: 10.3389/fpls.2021.693344 (PMC8264795; doi:10.3389/fpls.2021.693344)
Supplement: Supplementary file 1 [file Data_Sheet_1.pdf]

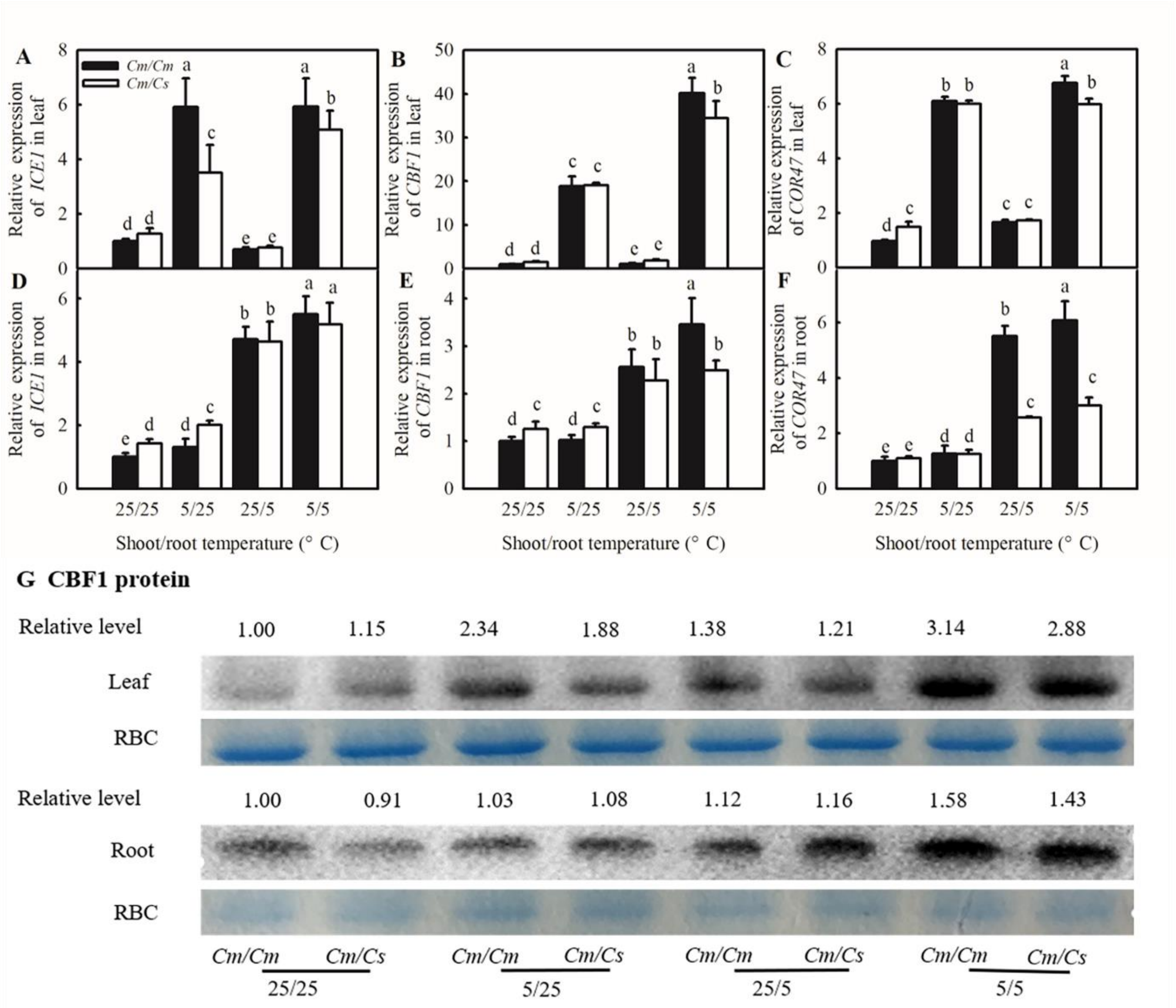

Supplementary Figure 1. Changes of the relative mRNA expression of *ICE*, *CBF1* and *COR47*, and the protein level of *CBF1* in the *Cm/Cm* and *Cm/Cs* plants under aerial or/and root-zone chilling stress. **(A)-(F),** Relative mRNA expression of *ICE1*, *CBF1* and *COR47* in leaves and roots; **(G)**, The protein level of *CBF1* in leaf and root. Three-leaf stage seedling were exposure to chilling stress for 48 h. Data are the means of four replicates (± SDs). Different letters indicate differ significantly between samples according to the Duncan's new multiple range test ($P < 0.05$).

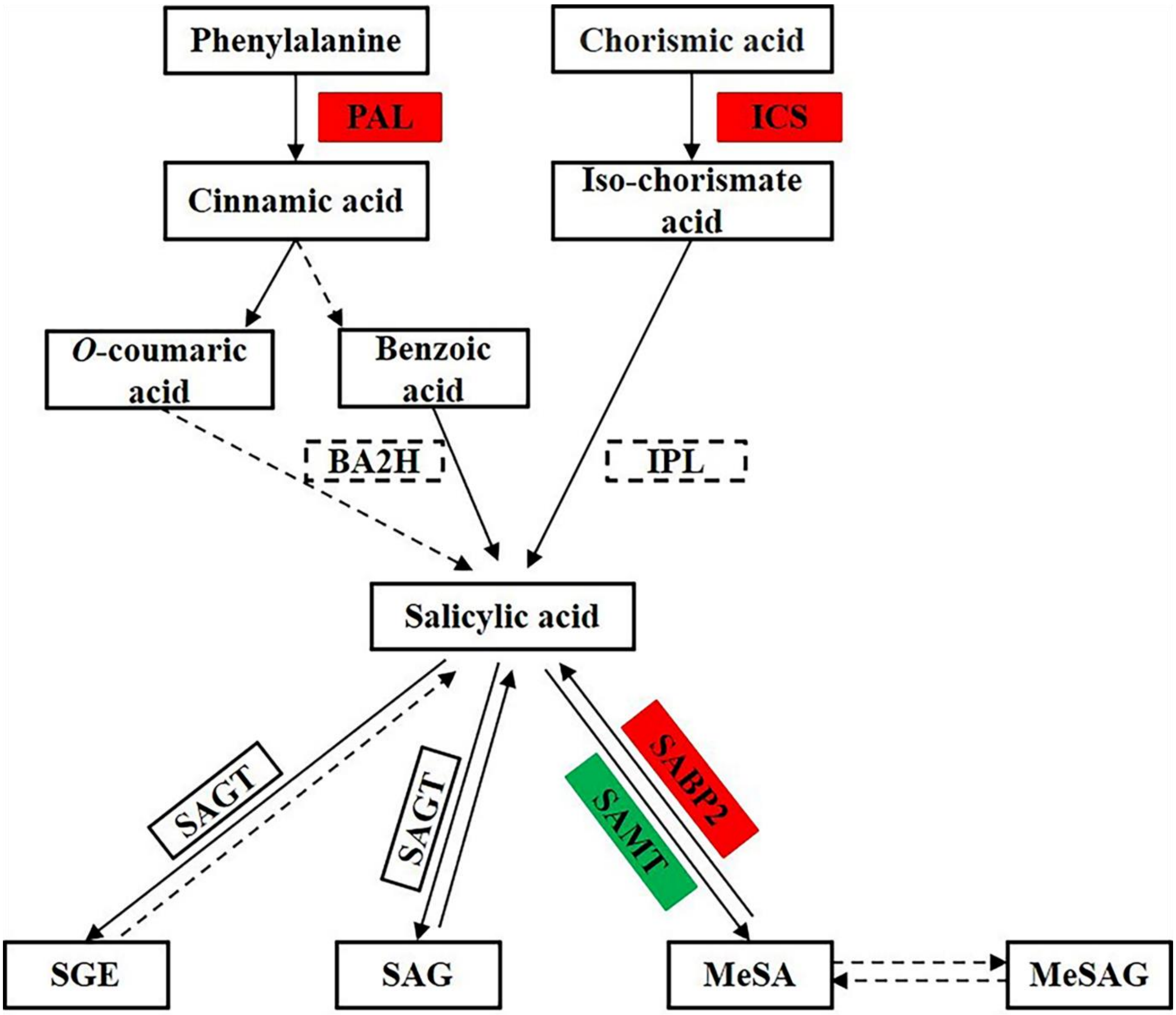

Supplementary Figure 2. Salicylic acid biosynthesis and metabolic pathways. Red indicates up-regulated genes, and green indicates down-regulated genes.

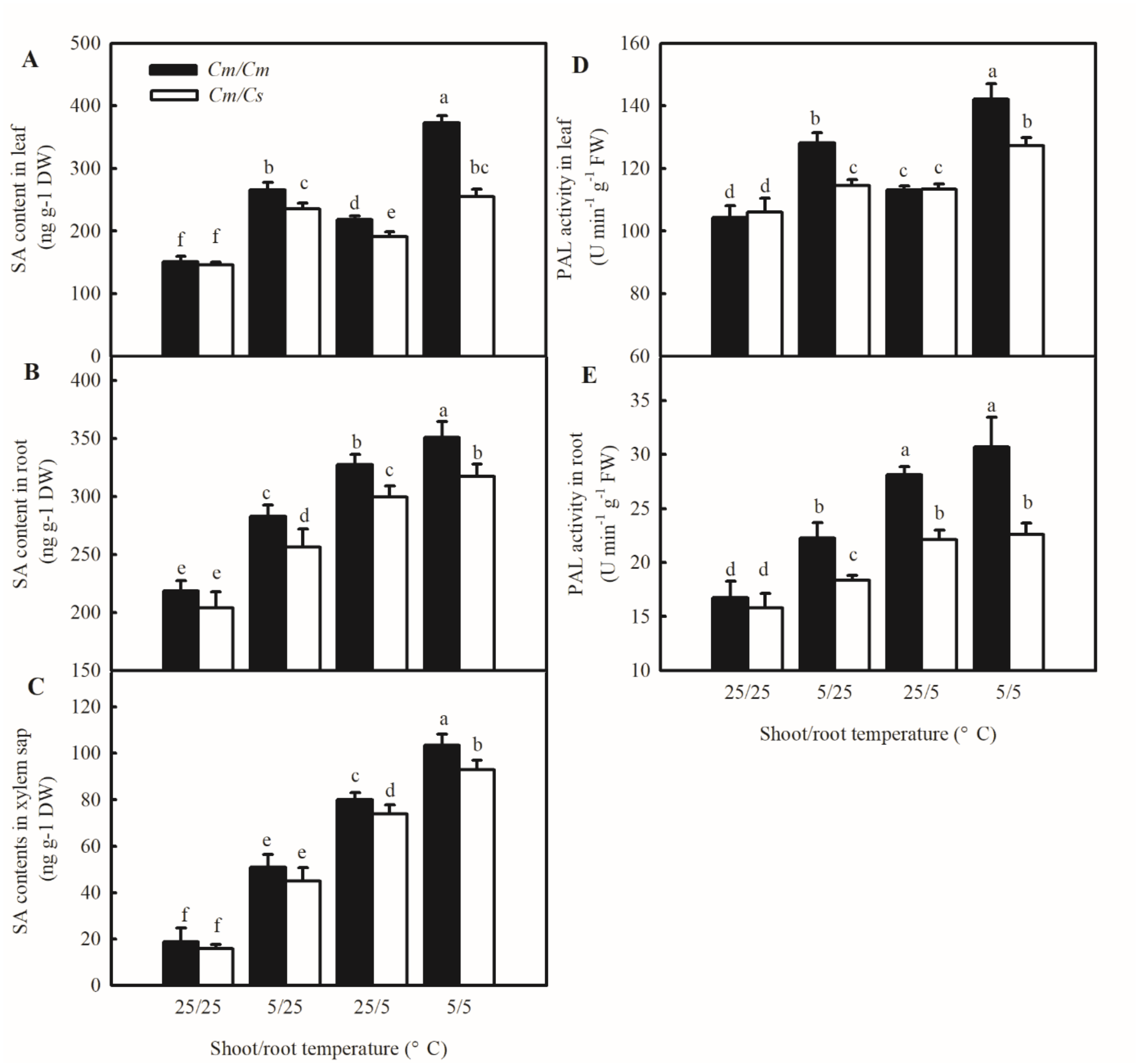

Supplementary Figure 3. SA biosynthesis and transport in response to rootstock and aerial or/and chilling stress in *Cm/Cm* and *Cm/Cs* plants. **(A)-(C),** SA content in leaves, roots and xylem sap respectively; **(D) and(E),** PAL activity in leaves and root respectively. The third leaf of three-leaf stage seedlings was sampled at 12 h after chilling treatment. Data are the means of four replicates (± SDs). Different letters indicate differ significantly between samples according to the Duncan's new multiple range test ($P < 0.05$).
